# Impact of pruritus in patients undergoing hemodialysis in Italy: a patient-based survey

**DOI:** 10.1007/s40620-024-01983-y

**Published:** 2024-06-24

**Authors:** Antonio Santoro, Dino Gibertoni, Andrea Ambrosini, Maria Elisabetta De Ferrari, Giuseppe Vanacore

**Affiliations:** 1 Casa di Cura Villa Toniolo, Via Toscana 34, 40141 Bologna, Italy; 2 Scientific Committee, ANED Associazione Nazionale Pazienti in Dialisi, Milan, Italy; 3 Epidemiology and Statistics Unit, IRCCS Azienda Ospedaliero-Universitaria di Bologna, Bologna, Italy; 4 ASST dei Sette Laghi, Varese, Italy; 5 Nefrologia, Dialisi e Trapianto, ASST Grande Ospedale Metropolitano Niguarda, Milan, Italy; 6 ANED Associazione Nazionale Pazienti in Dialisi, Milan, Italy

**Keywords:** Hemodialysis, Pruritus itching, Quality of life, Patient perspective

## Abstract

**Background:**

Itching is an annoying symptom which afflicts patients with chronic renal failure. We aimed to assess the impact and patient’s perception and experience of itching in the dialysis population in Italy.

**Methods:**

A questionnaire was developed by the National Hemodialysis and Dialysis Association of Italy (ANED) and administered to 996 hemodialysis recipients across 153 Italian dialysis centers. The main outcomes investigated by the questionnaire were patients’ satisfaction on answers regarding the nature of itching; continuing to talk about itching with the nephrologist; beliefs about resolution of itching.

**Results:**

A total of 1903 patients from 153 centers responded to the questionnaire. Patients who responded had a mean age of 67.9 ± 13.8 years (63.9% male) and were stratified by itch discomfort graded as mild (35.9%), moderate (29.6%), and severe (34.4%). Severe itching disrupted patients' daily lives, strained their relationships, caused anxiety, and diminished their quality of life. Patients with severe itch were more likely to talk about it with dialysis staff and to undertake dermatological visits. However, only 18.0% of patients reporting severe itching found the clinicians' responses satisfactory, compared to 49.1% of mild itch patients. Those who continued talking to nephrologists about itching received more satisfactory response. However, 40.8% believed itching could not be alleviated and were less likely to discuss it with nephrologists.

**Conclusions:**

There is an intricate relationship between the severity of itching, patient perceptions, and healthcare communication among hemodialysis patients. A substantial proportion of patients experiencing severe itching expressed feelings of resignation, highlighting the pressing need for enhanced clinician-patient communication.

**Graphical Abstract:**

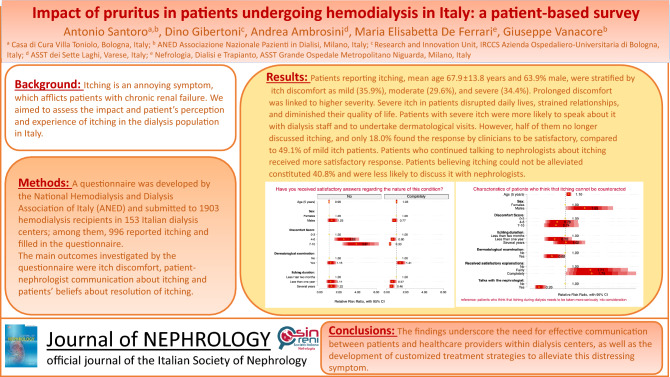

**Supplementary Information:**

The online version contains supplementary material available at 10.1007/s40620-024-01983-y.

## Introduction

Uremic pruritus is an extremely distressing symptom experienced by individuals undergoing dialysis. It significantly impacts upon their quality of life (QoL), disrupts sleep, and can even lead to organic disturbances in various bodily systems, occasionally resulting in severe depressive states. This itching, often referred to as uremic itching, has multiple underlying causes that extend beyond simple uremic intoxication [1, 2]. Factors such as disruption in calcium/phosphorus metabolism, nutritional deficiencies, nerve-related issues, and changes in skin moisture levels may all contribute to the root causes of pruritus. Dialysis adequacy also plays a role [3].

The incidence of itching in dialysis patients is reported to be higher than 20% [4]. Often, healthcare professionals tend to overlook itching, focusing more on what they consider more relevant clinical symptoms [3].

Typically, research on itching has heavily relied on the perspectives and observations of treating physicians, with limited attention given to the patients themselves. Only a few studies have explored the patients' viewpoints regarding the real-life impact of pruritus [5–8]. Among these, only the recent Dialysis Outcomes and Practice Patterns Study (DOPPS) in phases 4–6 (2009–2018) examined the effect of itching on the QoL of dialysis patients in Italy [9].

The objective of this Italian survey was to comprehensively evaluate the real-life impact of pruritus on individuals undergoing dialysis, directly from the patients' perspective. To achieve this, ANED (Associazione Nazionale Emodializzati e Dializzati) [10] developed a questionnaire distributed by patients to patients across multiple dialysis centers in Italy.

## Methods

### Study population

ANED invited 261 Italian dialysis centers across the country to involve their patients in the survey. Patients were eligible if they were under the care of a nephrologist and had given their consent to participate in the study.

### The survey

The questionnaire was designed with the help of patients who suggested some of the main questions through a focus group. It was distributed both in paper and online form directly to patients by their center representatives, who were themselves either patients undergoing dialysis or kidney transplant recipients and were active members of ANED. The research was limited to those centers where active representatives were present, and was carried out between November 23, 2021, and April 1, 2022. One week after distribution, the completed paper questionnaires, along with those completed online, were consolidated by ANED into a single database from which all statistical analysis was performed.

The questionnaire included queries on demographic data and 16 questions related to the intensity of itching, duration of itching, reporting to medical professionals, as well as any remedies suggested by various healthcare providers (Table 1S). To assess the intensity of itching, the Numeric Rating Scale (NRS) was utilized [11]: mild itching: scores 1–3; moderate itching: scores 4–6; severe itching: scores 7–10.

### Statistical analysis

Frequencies of response to each item were reported as mean and standard deviation (SD) or absolute frequency and percentage, overall and broken down by itch class. The Chi-square test was used to verify the hypothesis of response independence among itch classes. Four items related to patients’ behavior or perception were analyzed as dependent variables by multivariable binary or multinomial logistic regression models: #8 “Have you received satisfactory answers regarding the nature of this condition?”; #12 “Do you continue to talk about itching with your nephrologist?”; a combination of items #12 and #14 identifying patients who still talk with nephrologists and/or nurses; and #15 “He believes that the itching he suffers from …”. These models were analyzed sequentially, so that the last one included the previous models’ outcome variables as covariates; all models included itch class as main exposure, and age, sex, itching duration and dermatologist’s consultation as covariates. In all models, robust standard errors were obtained according to clustering of patients into dialysis centers. A 2-sided *p*-value of < 0.05 was considered statistically significant. All analyses were performed using Stata v.18.0 (StataCorp, College Station, Texas, USA).

## Results

A total of 1903 patients from 153 centers responded to the questionnaire. Among them, 1849 self-reported their itch score, with 897 (48.5%) assigning a score of 0 or 1. Most of these patients, with no or extremely mild itching, did not answer the following questions in the questionnaire, therefore the analysis was carried out on 996 (53.3%) patients who provided their itch score and at least one answer (Table 2S). The mean age was 67.0 ± 13.8 years and 636 (63.9%) patients were male (Table 1). Patients were stratified into 3 groups according to their reported level of itch discomfort: mild itch (*n* = 358, 35.9%); moderate itch (*n* = 295, 29.6%); severe itch (*n* = 343, 34.4%). No differences were observed across groups either between the number of females and males, or in the mean age of patients. Among the respondents, 234 (26.7%) reported that they had suffered from itching for less than 2 months, 266 (30.3%) for less than 1 year and 377 (43.0%) for several years (Table 1). The duration of discomfort went hand in hand with its intensity: those who reported suffering for less than 2 months were more frequently among those who indicated mild discomfort (42.5%), while conversely, those who had been suffering for several years made up the majority (53.3%) of those who described it as a very serious discomfort. When considering the affected body areas, 50.4% of patients reported experiencing itching on their back, 49.5% on their legs, 40.6% on both arms, and 28.6% on their chest (data not shown).

**Table 1:** Patient characteristics for the entire population and across itching severity categories, score ranges: 0–3 (mild itching), 4–6 (moderate itching), and 7–10 (severe itching)

	Total (*N* = 996)	0–3 (*N* = 358)	4–6 (*N* = 295)	7–10 (*N* = 343)	Test; *p*-value
Males	636 (63.9)	233 (65.1)	185 (62.7)	218 (63.6)	0.41; 0.81
Age (*n* = 989)	67.0 ± 13.8	67.7 ± 14.2	67.2 ± 13.3	66.2 ± 13.8	1.05; 0.35
2. How long have you been experiencing itching? (*N* = 877)					
Less than 2 months	234 (26.7)	121 (42.5)	61 (22.2)	52 (16.4)	66.43; < 0.001
Less than 1 year	266 (30.3)	86 (30.2)	84 (30.5)	96 (30.3)	
Several years	377 (43.0)	78 (27.4)	130 (47.3)	169 (53.3)	
4. How would you describe your chronic itching?(*N* = 943)					
It occurs only during dialysis	98 (10.4)	31 (9.5)	37 (12.8)	30 (9.1)	304.01; < 0.001
A mild discomfort	385 (40.8)	239 (73.3)	112 (38.9)	34 (10.3)	
It is present at night	171 (18.1)	19 (5.8)	45 (15.6)	107 (32.5)	
It is present for most of the day	191 (20.3)	27 (8.3)	67 (23.3)	97 (29.5)	
It is always present	98 (10.4)	10 (3.1)	27 (9.4)	61 (18.5)	
5. To what extent does your chronic itching affect your daily life? (*N* = 956)					
It limits me in my relationships with my family	19 (2.0)	0 (0)	5 (1.7)	14 (4.2)	240.46; < 0.001
It limits me with everyone else	99 (10.4)	7 (2.1)	27 (9.3)	65 (19.3)	
It makes me nervous, prevents me from sleeping	263 (27.5)	36 (10.9)	68 (23.5)	159 (47.3)	
It is just a disturbance that I easily overcome	575 (60.1)	288 (87.0)	189 (65.4)	98 (29.2)	
6. How did you manage the chronic itching at the beginning? (*N* = 908)					
I have talked to other patients in my center	20 (2.2)	5 (1.6)	7 (2.5)	8 (2.5)	70.71; < 0.001
I have used home remedies (e.g. cosmetics, cold shower)	268 (29.5)	134 (43.7)	72 (26.3)	62 (19.0)	
I have gone to the pharmacy to buy skin creams	154 (17.0)	56 (18.2)	48 (17.5)	50 (15.3)	
I have discussed it with my general practitioner	48 (5.3)	10 (3.3)	19 (6.9)	19 (5.8)	
I have talked about it immediately with the dialysis staff	319 (35.1)	86 (28.0)	92 (33.6)	141 (43.1)	
I have visited a dermatologist	78 (8.6)	8 (2.6)	29 (10.6)	41 (12.5)	
I don’t know	21 (2.3)	8 (2.6)	7 (2.5)	6 (1.8)	
8. Who among these told you that they suffer from chronic itching? (*N* = 763)					
Dermatologist	53 (7.0)	14 (5.3)	18 (7.9)	21 (7.7)	2.05; 0.9
My family doctor	43 (5.6)	15 (5.7)	13 (5.7)	15 (5.5)	
Nephrologist	525 (68.8)	184 (70.2)	152 (66.7)	189 (69.2)	
The dialysis nurse	142 (18.6)	49 (18.7)	45 (19.7)	48 (17.6)	
9. Have you received satisfactory answers about the nature of this condition? (*N* = 882)					
I haven't had satisfactory answers	233 (26.4)	31 (10.6)	71 (26.7)	131 (40.6)	98.83; < 0.001
I wouldn't know how to answer	24 (2.7)	9 (3.1)	7 (2.6)	8 (2.5)	
Yes, somewhat	339 (38.4)	109 (37.2)	104 (39.1)	126 (39.0)	
Yes, I have received complete and satisfactory responses	286 (32.4)	144 (49.1)	84 (31.6)	58 (18.0)	
10. Have you been seen by a dermatologist? (*n* = 924)					
No, I have never been seen by a dermatologist	656 (71.0)	259 (80.7)	187 (67.5)	210 (64.4)	25.45; < 0.001
Yes, I have decided to have a consultation	177 (19.2)	36 (11.2)	64 (23.1)	77 (23.6)	
Yes, I was referred by the nephrologist of my dialysis center	91 (9.8)	26 (8.1)	26 (9.4)	39 (12.0)	
13. Do you continue to talk about itching with your nephrologist? (*N* = 930)					
No	630 (67.7)	259 (80.2)	200 (70.9)	171 (52.6)	58.22; < 0.001
Yes	300 (32.3)	64 (19.8)	82 (29.1)	154 (47.4)	
14. If you answered no, can you explain why you don't talk about it? (*N* = 622)					
I realized that nothing can be done	469 (75.4)	183 (81.0)	151 (74.0)	135 (70.3)	7.56; 0.11
The nephrologist said we did everything we could	109 (17.5)	29 (12.8)	37 (18.1)	43 (22.4)	
It embarrasses me a lot	44 (7.1)	14 (6.2)	16 (7.8)	14 (7.3)	
15. Do you discuss itch with the dialysis nurse? (*N* = 885)					
I don't talk about it	414 (46.8)	165 (55.2)	125 (47.5)	124 (38.4)	32.11; < 0.001
Only occasionally	229 (25.9)	49 (16.4)	65 (24.7)	115 (35.6)	
Yes, he/she gives me useful advice	242 (27.3)	85 (28.4)	73 (27.8)	84 (26.0)	
16. He believes that the itching he suffers from (*N* = 846)					
It needs to be studied further	193 (22.8)	41 (15.0)	54 (20.6)	98 (31.6)	38.97; < 0.001
It needs to be taken seriously into consideration	345 (40.8)	101 (36.9)	114 (43.5)	130 (41.9)	
It cannot be counteracted; I must resign myself	308 (36.4)	132 (48.2)	94 (35.9)	82 (26.5)	

Data are presented as number and %. *N* represents the number of subjects

In response to the question “How would you describe your chronic itching to another patient with chronic kidney disease?”, the majority of patients who described itching as very bothersome reported that it was always present, or was present for most of the day (48.0%), or that it disrupted their nighttime sleep (32.5%). Only 9.1% of these patients mentioned that the itching occurred exclusively during dialysis. Correspondingly, most patients with severe itching noted that this condition had a significant impact on their social, familial, and professional relationships. It also heightened their anxiety and, notably, hindered their ability to sleep at night.

### Management of itching

As the level of itch increases from mild to severe, the percentage of patients who have discussed it immediately with dialysis staff and who have visited a dermatologist increases from 28.0 to 43.1%, and from 2.6 to 12.5%, respectively (Table 1). Conversely, there was a significant reduction in the proportion of those who have used home-based remedies (from 43.7 to 19.0%). Consistently, patients with the greatest discomfort from chronic itching were significantly more frequently seen by a dermatologist, either as their own choice (23.6%) or prompted by the nephrologist (12.0%) than those who reported mild itch (11.2 and 8.1%, respectively). Regardless of the degree of discomfort from itching, it was the nephrologist and the dialysis nurse, in the vast majority of cases, who informed the patient about the chronicity of the itching in patients of all three classes of itching (Table 1).

Among the treatments used to alleviate itching, 48.4% of patients reported using emollient creams, 20.2% oral antihistamines, 10.2% cortisone-containing creams, 6.2% antihistamine-containing creams, and 3.5% medications prescribed by the nephrologist. The remaining patients stated that they used other remedies, including pregabalin, oral corticosteroids, injectable antihistamines, other medications prescribed by the dermatologist, anxiolytics, gabapentin, UVB radiation, and antidepressants, with percentages ranging from 1.9 to 0.8%.

### Awareness and individual perception

The patients’ level of satisfaction regarding the responses received from clinicians concerning the nature of itching was inversely associated with the degree of discomfort. Specifically, 49.1% of patients with mild symptoms found the response to be satisfactory. In contrast, among patients experiencing severe discomfort, only 18.0% expressed satisfaction with the response received, while 40.6% deemed them unsatisfactory (Table 1).

**Fig. 1:**
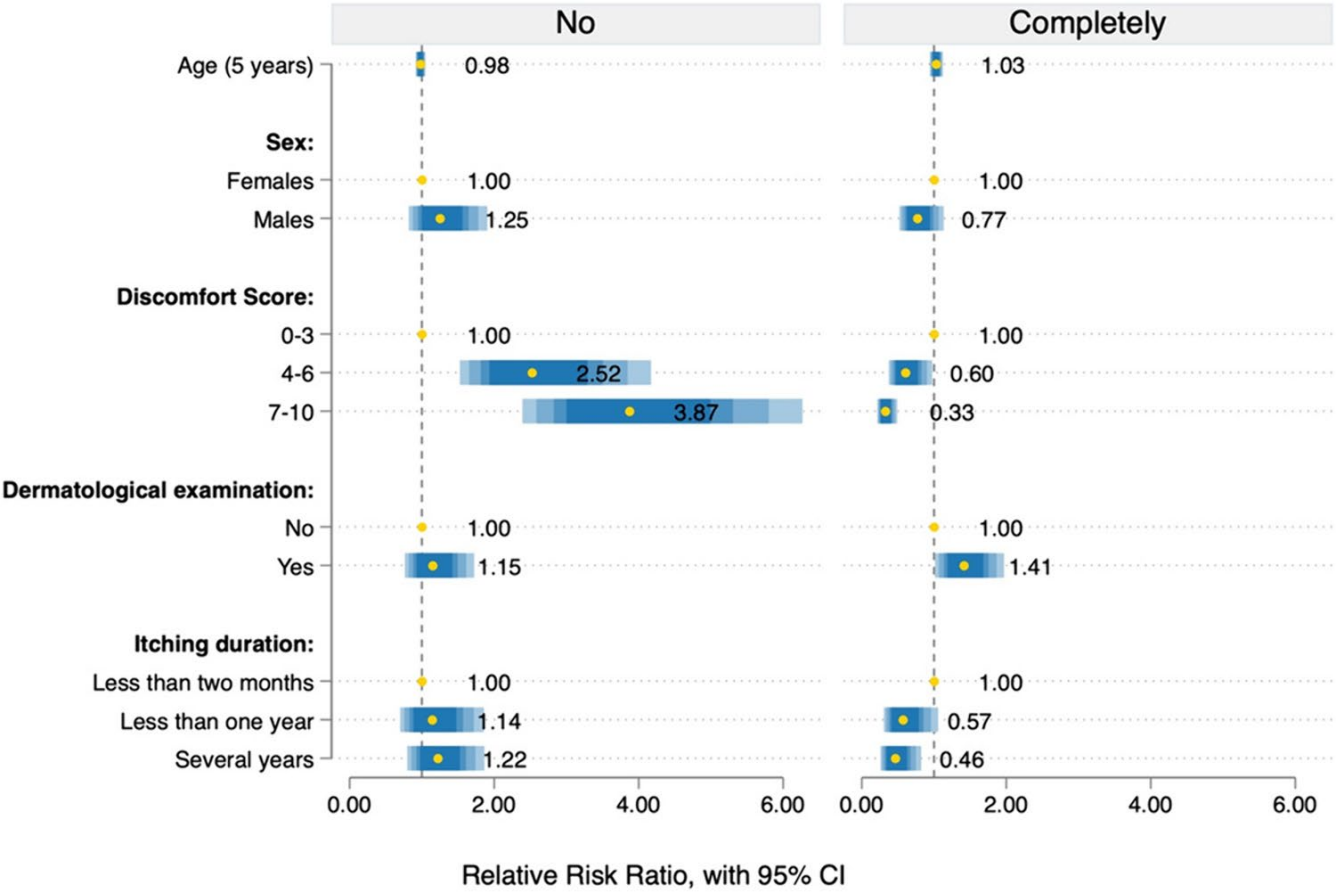
Multinomial logistic regression model, with respondents' answers to the question “Have you received satisfactory answers regarding the nature of this condition?” as the dependent variable. In this context, “no” signifies “I haven't received satisfactory answers,” while “completely” indicates “Yes, I have received comprehensive and satisfying answers.” Data are presented as relative risk ratios and 95% confidence intervals

Utilizing a multinomial logistic regression model, we found that patients with discomfort scores 4–6 and 7–10 had a 2.52 (*p* < 0.001) and 3.87 (*p* < 0.001) relative risk, respectively, to receive unsatisfactory responses when compared to patients with mild symptoms (Fig. 1). Conversely, the probability of receiving complete and satisfactory responses decreased for patients with discomfort scores 7–10 (RRR = 0.33, *p* < 0.001), those with scores 4–6 (RRR = 0.60, *p* = 0.04), and individuals who had been experiencing itching for several years (RRR = 0.46, *p* = 0.01) (Fig. 1).

### Communication with healthcare personnel

Regarding the response to the question, 'Do you continue to talk about itching with your nephrologist?' it emerged that a higher percentage of patients experiencing strong discomfort (47.4%) continued to discuss itching with their nephrologist, in contrast to those with moderate (29.1%) or mild discomfort (19.8%) (Table 1). Nevertheless, half of patients with scores ranging from 7 to 10 no longer discussed their itching. It must be noted that those who continued to talk to their nephrologist about itching had a more satisfactory response about the nature of the itching, unlike those who declared strong discomfort (data available on demand).

A multiple logistic regression model showed that the patients' likelihood of still discussing their itching with the nephrologist was 4.35 (*p* < 0.001) and 1.81 (*p* = 0.001) for patients with discomfort scores of 7–10 and 4–6, respectively, compared to those with scores of 0–3. Furthermore, undergoing a dermatological visit and providing complete and satisfactory answers about the nature of the itching more than doubled the likelihood (OR = 2.45, *p* < 0.001 and 2.25, *p* = 0.002, respectively) of still discussing their itching, compared to patients who did not have a dermatological visit and satisfactory answers.

Three hundred ninety-five (41.4%) patients reported not discussing their itching with either the nephrologist or the nurse (Table 2). Among them, 44.0% (*n* = 172) reported a low level of discomfort and for the majority of them, itching was just a disturbance (70.5%) that did not prevent them from carrying out their daily activities (Table 2). Only 27.4% said they had not received satisfactory answers regarding the nature of the itching, and 83.1% had become convinced that there was nothing that could be done about it. However, more than half of them believed that itching should be studied more thoroughly and taken more seriously into consideration (Table 2).

**Table 2:** Patient responses for the entire population and categorized based on their interactions with nurse, nephrologist, both, or neither

	Total (*N* = 954)	Does not talk to anyone (*N* = 395)	Talks only to the nephrologist or nurse (*N* = 422)	Talks to both of them (*N* = 137)	Test; *p*-value
Males	608 (63.7)	256 (64.8)	264 (62.6)	88 (64.2)	0.46; 0.79
Age (*N* = 871)	66.9 ± 13.9	66.3 ± 14.0	66.5 ± 13.8	69.9 ± 13.8	4.34; 0.03
Itch classes (*N* = 866)					
0–3	328 (34.8)	172 (44.0)	126 (30.3)	30 (22.2)	40.3; < 0.001
4–6	283 (30.0)	122 (31.2)	121 (29.1)	40 (29.6)	
7–10	331 (35.1)	97 (24.8)	169 (40.6)	65 (48.2)	
How long have you been experiencing itching? (*N* = 877)					
Less than 2 months	207 (26.3)	84 (28.2)	88 (24.5)	35 (26.9)	3.41; 0.76
Less than 1 year	238 (30.2)	86 (28.9)	108 (30.1)	44 (33.9)	
Several years	342 (46.5)	128 (42.9)	163 (45.4)	51 (39.2)	
To what extent does your chronic itching affect your daily life? (*N* = 858)					
It limits me in my relationships with my family	20 (2.2)	4 (1.1)	7 (1.7)	9 (6.8)	44.8; < 0.001
It limits me with everyone else	96 (10.3)	32 (8.4)	46 (11.1)	18 (13.5)	
It makes me nervous, prevents me from sleeping	256 (27.6)	76 (20.0)	139 (33.5)	41 (30.8)	
It is just a disturbance that I easily overcome	556 (59.9)	268 (70.5)	223 (53.7)	65 (48.9)	
Have you received satisfactory answers about the nature of this condition? (*N* = 818)					
I haven't had satisfactory answers	232 (26.5)	90 (27.4)	119 (28.9)	23 (17.0)	26.1; < 0.001
I wouldn't know how to answer	23 (2.6)	17 (5.2)	5 (1.2)	1 (0.7)	
Yes, somewhat	334 (38.2)	127 (38.7)	156 (38.0)	51 (37.8)	
Yes, I have received complete and satisfactory responses	285 (32.6)	94 (28.7)	131 (31.9)	60 (44.4)	
If you don’t talk of itch with nephrologist, can you explain why? (*N* = 603)					
I realized that nothing can be done	468 (75.2)	250 (83.1)	191 (71.0)	27 (51.9)	46.9; < 0.001
The nephrologist said we did everything we could	110 (17.7)	26 (8.6)	61 (22.7)	23 (44.2)	
It embarrasses me a lot	44 (7.1)	25 (8.3)	17 (6.3)	3 (3.9)	
He believes that the itching he suffers from (*n* = 803)					
It does not need to be studied further	190 (22.5)	46 (14.3)	104 (26.5)	40 (30.5)	74.1; < 0.001
It needs to be taken seriously	309 (36.6)	174 (54.2)	110 (28.0)	25 (19.1)	
It cannot be counteracted; I must resign myself	346 (40.9)	101 (31.5)	179 (45.5)	66 (50.4)	

Data are presented as number and %. *N* represents the number of subjects

Using a multiple logistic regression model, we found that individuals who believed that itching could not be alleviated, those who had not received satisfactory explanations, and those who had not undergone a dermatological visit, had a more than three-fold (OR = 3.02, *p* < 0.001), two-fold (OR = 2.11, *p* = 0.003), and almost two-fold (OR = 1.73, *p* = 0.004) likelihood of refraining from discussing it with anyone, respectively. In contrast, this behavior was less likely among patients with severe discomfort (OR = 0.43, *p* < 0.001) and older patients (OR = 0.93, *p* = 0.005).

**Fig. 2:**
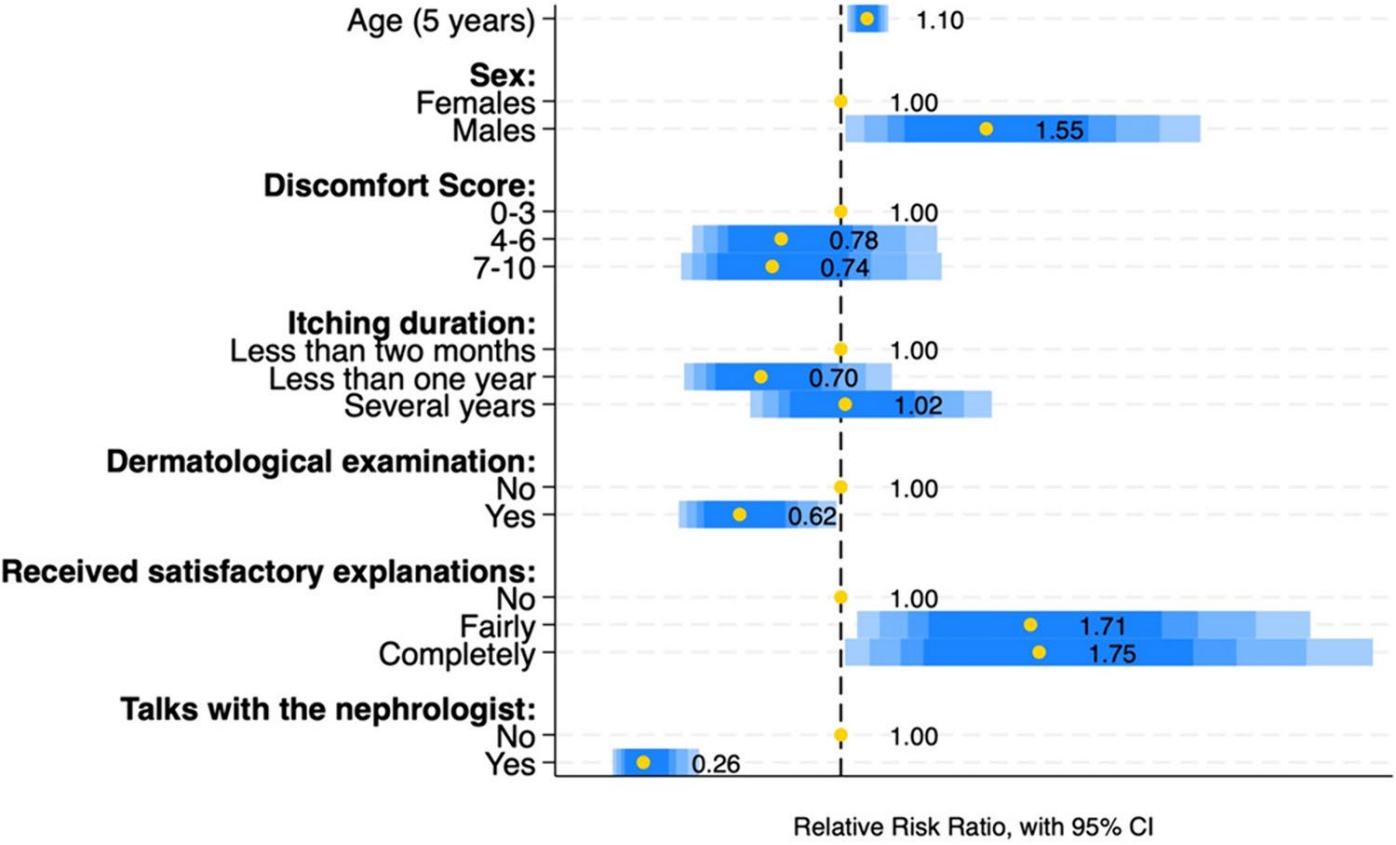
Multinomial logistic regression model to investigate whether the characteristics of those who think that itching cannot be counteracted (the “resigned”) can be associated with patients' perception of itch. The reference group was that of patients who think that itching during dialysis needs to be taken more seriously into consideration. The third possible answer to that question (itching during dialysis needs to be studied more thoroughly) was not included in order to obtain a tidier graph. Relative risk ratios are reported

The percentage of patients who believe that itching cannot be alleviated decreased as the reported discomfort increased, from 48.2% among those reporting mild discomfort to 26.5% among those with severe discomfort (Table 1). In the latter group, the most agreed upon opinion was that itching during dialysis needs to be taken seriously into consideration. Among patients who believed that itching cannot be alleviated, a large majority (83.1%) did not discuss itching with the nephrologist. However, patients who discussed their itching with the nephrologist were the ones most convinced that itching needs to be studied more thoroughly (and conversely, they were the least 'resigned'), and this was observed in all intensity groups of itching. Moreover, using multinomial logistic regression analysis we found that patients who thought that itching cannot be counteracted, referred to as “the resigned”, compared to those who think that itching during dialysis needs to be studied more thoroughly, were less likely to have spoken to the nephrologist (RRR = 0.26, *p* < 0.001), and to have had a dermatological visit (RRR = 0.62, *p* = 0.04). They were more likely to be male (RRR = 1.55, *p* = 0.04), older (RRR = 1.10, *p* = 0.01) and to have had fairly (RRR = 1.71, *p* = 0.03) or completely satisfactory responses (RRR = 1.75, *p* = 0.04) (Fig. 2). In particular, among those who think that the itching cannot be counteracted, and who declared the itching to be seriously bothersome (*n* = 82), just over half (*n* = 42) declared that they no longer talked about their itching either to the nephrologist or to the nurse. Only 8 (9.8%) continued to speak with both professionals.

## Discussion

In this patient-centered survey, we provide a unique insight into the impact of pruritus on the real lives of hemodialysis (HD) patients. The study involved 996 patients categorized into three groups based on the severity of their itching: mild itch (35.9%), moderate itch (29.6%), and severe itch (34.4%). Notably, the intensity of itching was significantly positively associated with longer duration, diminished QoL, interpersonal difficulties, sleep disturbances, and consultations with both dialysis staff and dermatologists. Severity of itching was inversely related to having satisfactory responses about the nature of itching. Subjects who no longer engaged in discussions with their nephrologist included individuals with severe itching and those who did not experience satisfactory responses. These patients can be considered resigned, comprising approximately 50% of those with severe itching.

The occurrence of pruritus in dialysis patients is quite common within this population, and is estimated to be approximately 40% [12]. Recently, Sukul et al. reported a frequency of mild pruritus at 30% and moderate to severe pruritus at about 37% [9], values closely aligned with ours. Nevertheless, there has been significant variability in the reported occurrence rates among different dialysis centers, ranging from 7 to 44% for moderate to severe itching [12]. This variation in frequency data may be influenced by the methods of data collection, which could include verbal information and questionnaires, the perspectives of dialysis staff, or the visibility of only the most bothersome symptoms, among other factors.

In this study, we observed that severe itching had a significant negative impact on various aspects of patients' daily lives, including their social, familial, and professional relationships, leading to increased anxiety. Analogously, the DOPPS study of more than 6000 hemodialysis patients across 17 countries from 2012 to 2015 found that among patients who reported being very much or extremely bothered by itching, 58% reported being depressed about the itching, 45% reported the itching made it hard to work, and 35% reported the itching reduced their desire to be with other people [4].

We observed that as the severity of itching increased from mild to severe, there was a corresponding increase in the percentage of patients seeking help from healthcare professionals, including dialysis staff and dermatologists. Recently, Santos-Alonso et al. [3] reported that dialysis personnel, both nursing and medical staff, remain the primary resource for patients when it comes to explaining the causes of itching and suggesting remedies [3]. Unfortunately, prescriptions provided by nephrologists have often proven to be ineffective. What emerges from our data is that patients tend to resign themselves to itching and lack trust in the responses of professionals and in available treatment options. In light of this, Aresi et al. [5] identified three main themes that shed light on the factors influencing whether patients communicate their itch-related concerns to medical personnel: understanding the underlying causes and available treatments for itch (including the lack of awareness regarding the link between itch and chronic kidney disease, as well as limited knowledge of potential treatment options); shaping attitudes concerning the significance of itch as a health-related issue, encompassing the perspectives of both patients and healthcare providers; establishing effective mechanisms to prompt itch assessment during medical consultations, involving routine practices, recognizing itch as a diagnostic marker, and evaluating the severity of itch symptoms.

Due to the concern for treatment of pruritus, the vast majority of our patients were using emollient creams and antihistamines. Antihistamines have traditionally been the most widely used therapeutic option, primarily as a first-line treatment, despite evidence from various studies indicating that they are not effective in reducing itching [13]. Unfortunately, the pathophysiological mechanisms involved in pruritus are multiple, complex, and largely unknown. The primary mechanisms can be grouped into four main hypotheses: uremic toxin deposition, dysregulation of the opioid system, immune system dysfunction, and peripheral neuropathy [14–16]. Uremic toxin accumulation and deposition have traditionally been associated with pruritus, as improving dialysis efficiency and reducing serum calcium, parathormone, or phosphorous levels have alleviated itching in some patients. Immune system dysregulation is still considered a potential contributor to itching as increased levels of eosinophils, mast cells, histamine, and tryptase have all been reported [15]. Inflammation is believed to sensitize small nerve fibers in the skin, leading to itching. Furthermore, high levels of markers of systemic inflammation are observed in patients with itching, including high levels of T cells, white blood cells, C-reactive protein, interleukins -6 and -2, and ferritin, alongside low levels of albumin [16]. Peripheral neuropathy, which is prevalent in dialysis patients, can cause itching when affected neurons are activated in the presence of pruritogens [16]. The dysregulation of the opioid system is a hypothesis gaining prominence. It is suggested that CKD involves an imbalance between OPRM1/μ-opioid receptor (MOR) and OPRK1/κ-opioid receptor (KOR), with an imbalance in favor of the MOR. These receptors are part of the endogenous opioid system and are used for the transmission of pain stimuli in a complex and peripheral system of neurons. Furthermore, these opioid receptors are responsible for the regulation of chronic itch [17]. Changes in the peripheral opioid system may play an important role in the pathogenesis of uremic itch. Wieczorek et al. reported a decrease of KOR expression in pruritic HD patients and a negative association between itch severity and KOR expression in the epidermis of HD patients [18]. In contrast to KOR, there is the possibility that peripheral MOR induces pruritus [19]. Pruritus can be increased via μ-receptor activation or κ-receptor blockade and decreased via κ-receptor activation or μ-receptor blockade [14]. Emerging therapies such as difelikefalin and other potential agents coming to market are focused on regulating the opioid system. Several drugs have been studied for pruritus treatment, including nalfurafine, nalbuphine, and difelikefalin. Nalfurafine is approved in Japan for refractory itching but not in Europe due to inconsistent results [20]. Nalbuphine has shown some promise in improving pruritus in hemodialysis patients, with no significant adverse events reported [11]. Difelikefalin, a highly selective peripheral KOR agonist, has shown significant results in reducing symptoms and improving QoL in patients suffering from pruritus [21]. Considering the promising clinical trial results, the possibility of medical treatment with difelikefalin under compassionate use was assessed. Difelikefalin has received approval from the United States Food and Drug Administration (FDA) and the European Medicines Agency (EMA) for use in hemodialysis patients with pruritus, although it is not yet available in Europe. It appears relatively safe and effective, and once it receives marketing authorization, it may become the preferred treatment for moderate-to-severe itching in dialysis patients, along with topical emollient or moisturizing cream application. Nonetheless, 20% of severely affected patients in the large DOPPS cohort were not treated for itch [9], reflecting low treatment rates found in other studies [22, 23]. But what is even more concerning is that Sukul et al. reported that 91% of patients in Italy were not treated, with only 4% receiving antihistamines and 4% receiving gabapentin and pregabalin [9], even though new treatments are available, as described above.

This study is subject to several limitations: first, the absence of linkage with the patients’ clinical data, especially concerning time since dialysis initiation and the dialysis procedure employed, and dialysis efficiency, which could influence the severity of their itching, as well as their behaviors and perceptions. These data were not requested from patients as it would have likely induced a recall bias; moreover, we anticipated that it would not have been feasible due to privacy regulations to obtain these data from all dialysis centers. However, in hindsight, we acknowledge that simple information such as when dialysis treatment was initiated, the type of dialysis and the frequency of treatment, could have been added. Another limitation stems from the questionnaire used in this study, which was created based on patients' suggestions. While this approach is indeed valuable, it may introduce a limitation in terms of questionnaire validation, potentially affecting the reliability of the data obtained. On a similar note, it was reported that some patients were unable to independently complete the entire questionnaire due to the complexity of some items. Finally, due to the voluntary nature of the study participation, we were unable to establish to what extent our results are representative across all dialysis patients. By addressing these limitations in subsequent studies, we can strive to improve our understanding in this area and yield more reliable and widely applicable findings.

## Conclusions

This large Italian patient-centered survey explores the multifaceted challenges of pruritus as experienced by dialysis patients in their everyday lives. The findings highlight the critical need for effective communication between patients and healthcare providers within dialysis centers, as well as the development of customized treatment strategies to alleviate this distressing symptom. Further research is warranted to enhance our comprehension and improve the management of itching in this particular context.

## Supplementary Information

Below is the link to the electronic supplementary material.

Supplementary file1 (DOCX 17 KB)

## Data Availability

The datasets generated during and/or analysed during the current study are available in the figshare repository, https://figshare.com/s/37f5f9ae608293c50aee.
