# Correction to: Impact of pruritus in patients undergoing hemodialysis in Italy: a patient‑based survey

**DOI:** 10.1007/s40620-024-02028-0

**Published:** 2024-07-13

**Authors:** Antonio Santoro, Dino Gibertoni, Andrea Ambrosini, Maria Elisabetta De Ferrari, Giuseppe Vanacore

**Affiliations:** 1 Casa di Cura Villa Toniolo, Via Toscana 34, 40141 Bologna, Italy; 2 Scientific Committee, ANED Associazione Nazionale Pazienti in Dialisi, Milan, Italy; 3 Epidemiology and Statistics Unit, IRCCS Azienda Ospedaliero-Universitaria di Bologna, Bologna, Italy; 4 ASST dei Sette Laghi, Varese, Italy; 5 Nefrologia, Dialisi e Trapianto, ASST Grande Ospedale Metropolitano Niguarda, Milan, Italy; 6 ANED Associazione Nazionale Pazienti in Dialisi, Milan, Italy

**Correction to: Journal of Nephrology** 10.1007/s40620-024-01983-y

In this article the statement in the Funding information section was incorrectly given as 'Open access funding provided by Alma Mater Studiorum - Università di Bologna within the CRUI-CARE Agreement. No funding was received for conducting this study.' and should have read 'Open access funding provided by Alma Mater Studiorum - Università di Bologna within the CRUI-CARE Agreement. ANED thanks CSL Vifor for the financial support for the realization of this study'.

The original article has been corrected.

