# Supplementary material for: Impact of pruritus in patients undergoing hemodialysis in Italy: a patient-based survey
Source: J Nephrol. 2024 Jun 24;37(7):1957–66. doi: 10.1007/s40620-024-01983-y (PMC11519116; doi:10.1007/s40620-024-01983-y)
Supplement: Supplementary file 1 — Supplementary file1 (DOCX 17 KB) [file 40620_2024_1983_MOESM1_ESM.docx]

**Impact of pruritus in patients undergoing pruritus**

**hemodialysis in Italy: a patient-based survey**

Antonio Santoro, Dino Gibertoni, Andrea Ambrosini, Maria Elisabetta De Ferrari, Giuseppe Vanacore

**Supplementary materials**

**Table 1S**. List of survey items

| **Nr** | **Items** |
| --- | --- |
| 1 | To what extent have you been bothered by itchy skin in the last two months? |
| 2 | How long have you been experiencing itching? |
| 3 | Which body parts are affected by pruritus? |
| 4 | How would you describe your chronic itching to another patient with chronic kidney disease |
| 5 | To what extent does your chronic itching affect your daily life? |
| 6 | How did you manage the chronic itching at the beginning? |
| 7 | When you decided to contact a doctor at the dialysis center, who did you initially talk to about it? |
| 8 | Who among these told you that they suffer from chronic itching? |
| 9 | Have you received satisfactory answers about the nature of this condition? |
| 10 | Have you been seen by a dermatologist? |
| 11 | Did you receive further information if you visited the dermatologist? |
| 12 | Thinking about all the treatments you have undergone so far to relieve your itching, including non-pharmacological ones, can you list them? |
| 13 | Do you continue to talk about itching with your nephrologist? |
| 14 | If you answered no, can you explain why you don't talk about it? |
| 15 | Do you discuss itch with the dialysis nurse? |
| 16 | He believes that the itching he suffers from: needs to be studied... More |

**Table 2S.** Number of patients in each itching severity class who responded to at least one question.

| **Itching Scores** | **Total** | **At least 1 question answered** |
| --- | --- | --- |
| 0 | 439 | 12(2.7%) |
| 1 | 458 | 131(28.6%) |
| 2 | 138 | 109(79.0%) |
| 3 | 122 | 106(86.9%) |
| 4 | 100 | 88(88.0%) |
| 5 | 134 | 119(88.8%) |
| 6 | 93 | 88(94.6%) |
| 7 | 108 | 103(95.4%) |
| 8 | 135 | 126(93.3%) |
| 9 | 36 | 30(83.3%) |
| 10 | 86 | 84(97.7%) |
| Total | 1849 | 996(53.9%) |

Data are presented as number and %.
